# Optimization of Ultrasound‐Assisted Methanolic Extraction of *Terminalia arjuna* Bark Using Response Surface Methodology and Characterization of the Extract

**DOI:** 10.1002/fsn3.71232

**Published:** 2025-11-26

**Authors:** Hafsa Tahir, Muhammad Nadeem Akhtar, Hamid Saeed, Muhammad Jawad, Muhammad Afzaal, Manashree, Apurav Gautam, Fakhar Islam, Catherine Tamale Ndagire

**Affiliations:** ^1^ University Institute of Diet and Nutritional Sciences The University of Lahore Lahore Pakistan; ^2^ Department of Nutrition and Dietetics, School of Health Sciences University of Management and Technology Lahore Pakistan; ^3^ Punjab University College of Pharmacy University of Punjab Lahore Pakistan; ^4^ Department of Public Health Health Services Academy Islamabad Pakistan; ^5^ Department of Food Science Government College University Faisalabad Pakistan; ^6^ Department of Forensic Science, School of Sciences JAIN (Deemed to Be University) Bangalore Karnataka India; ^7^ Centre for Research Impact & Outcome Chitkara University Institute of Engineering and Technology, Chitkara University Rajpura Punjab India; ^8^ Sharda School of Pharmacy Sharda University Greater Noida India; ^9^ Institute of Food Sciences Nur International University Lahore Pakistan; ^10^ Department of Food Innovation and Nutrition Mountains of the Moon University Fort Portal Uganda

**Keywords:** Box–Behnken design, medicinal plants, methanol extract, phytochemicals, proximate analysis, terminalia arjuna bark, ultrasound‐assisted extraction

## Abstract

*Terminalia arjuna*'s bark is a renowned medicinal plant material within the Indian subcontinent. Ultrasound‐assisted extraction (UAE) of *Terminalia arjuna* bark was optimized using response surface methodology (RSM) with methanol as the extraction solvent. Methanol was selected for its ability to efficiently solubilize phenolic and flavonoid compounds, which are major contributors to the bark's bioactivity. A Box–Behnken design was followed to model the effects of temperature, solvent‐to‐solid ratio, extraction time and sonication power on the yield. The optimized UAE conditions produced an extract with a yield of 12.09%, which was highly enriched in phenolic (442.2 ± 16.2 mg GAE/g) and flavonoid (294.6 ± 8.1 mg QE/g) compounds, demonstrating the method's efficiency for targeted phytochemical recovery. Proximate and mineral analysis revealed the bark's nutritional composition and its content of essential minerals like calcium (86.27 ± 1.43 mg/kg) and iron (16.03 ± 1.21 mg/kg). The extract exhibited significant in vitro antioxidant activity, with an IC₅₀ of 186.4 μg/mL in the DPPH assay and 24.4 μM Fe (II) equivalents in the FRAP assay. The study demonstrates that UAE is an efficient technique for obtaining a phytochemically rich extract from *T. arjuna* bark, which shows antioxidant potential.

## Graphical abstract

*Terminalia arjuna*'s bark is a renowned medicinal plant material within the Indian subcontinent. This study sought to enhance the ultrasound‐assisted extraction (UAE) technique **t**o maximize the extraction yield from *T. arjuna* bark using Response Surface Methodology (RSM).

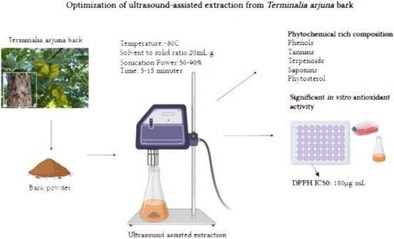

## 1 Introduction

Plants have been commonly utilized as an exceptional source in the manufacturing of medicine. Throughout human history, human civilization took multiple benefits from plants that play a prominent role in the treatment of diseases. Traditionally, the alternative approach to producing medicines resulted in the production of more than 85% of drugs through plant‐based resources (Kaushik et al. 2021). While many medicinal plants have been utilized historically to treat an assortment of ailments, today's modern medical system uses very few plant‐based products to cure most illnesses, including skin disorders, diabetes cough, ulcers, increased sweating, tumors, asthma, and cardiovascular diseases (CVD) (Prabhakar and Banerjee 2020). One of the subcontinent's native medicinal trees is *Terminalia arjuna* (Roxb.) Wight and Arn., or *T. arjuna*, more often referred to as “Arjuna” (Gaikwad and Jadhav 2018). *T. arjuna* is a tall, evergreen deciduous tree native to the subcontinent, belonging to the Combretaceae family. It typically reaches heights of 20 to 30 m. This tree is a crucial component in numerous Ayurvedic formulations aimed at enhancing cardiovascular health. It is recognized for its hypocholesterolemic, and antioxidant properties (Amalraj and Gopi 2017), as demonstrated by multiple in vivo studies conducted on both animal and human subjects. Additionally, the plant has shown potential anticancerous and antibacterial actions (Khatkar et al. 2019). Numerous beneficial phytoconstituents have been extracted from *T. arjuna*. The main reason for the cardiovascular effects is the changes brought about by triterpenoids. Its anticancer qualities are attributed to flavonoids and tannins (Kumar et al. 2023). The antioxidant activity of *T. arjuna* is assessed by measuring the functioning of glutathione S‐transferase, superoxide dismutase and catalase which are essential elements of the antioxidant defense mechanism that help eliminate reactive oxygen species (ROS) (Amalraj and Gopi 2017). Oral delivery of various dosages of *T. arjuna* aqueous extract significantly boosts the activities of S‐transferase catalase, superoxide dismutase, and glutathione. Furthermore, *T. arjuna* diminishes anaerobic metabolism by decreasing lactate dehydrogenase activity (Hebbani et al. 2021; Khatkar et al. 2019). *T. arjuna's* various components have been investigated and discovered to have antibacterial, antimicrobial, antitumoral, antifeedant, antiallergic, anti‐HIV, and antifertility properties. They are also used to treat fractures, ulcers, and hepatic disorders (Awad et al. 2022). The inotropic effects of *T. arjuna* are considered to be attributed to its saponin glycosides, whereas the flavonoids/phenolics may have vascular amplification and antioxidant properties (Yang et al. 2024). This demonstrates the plant's various cardio‐protective effects (Ramesh and Palaniappan 2023).

Various techniques for extracting plant materials have been documented for centuries, and researchers have developed many newer methods to improve these processes. Ultrasound‐assisted extraction, supercritical fluid extraction and microwave‐assisted extraction are considered innovative techniques; meanwhile room temperature extraction, Soxhlet extraction, and decoction are classified as traditional extraction techniques (AOAC International 2020). Phytochemical components including flavonoids, phenolics, tannins, saponins and terpenoids are being determined in the extracts of *T. arjuna* bark under ideal ultrasonic‐assisted extraction (UAE) conditions (Paul et al. 2022). Ultrasound‐assisted extraction has become significant among innovative extraction procedures due to the power of ultrasound waves to penetrate deeply into cells, resulting in rupture and enhanced extraction efficiency (Chemat et al. 2017). Ultrasonic waves with a high frequency are used in ultrasound‐assisted extraction (UAE) to create acoustic cavitation or vesicles around the cells, causing their rupture. This leads to the breaking of cell membranes, allowing the discharge of intracellular contents and escalating the rate of mass transfer. The method is easy to operate, cost‐effective, and can be scaled up easily, although the initial capital investment can be prohibitive. It requires minimal energy and operates at low temperatures, which helps preserve the activity of bioactive compounds. UAE is increasingly utilized for extracting carotenoids, aromas, polysaccharides and polyphenols from vegetative biomass (Tiwari 2015). Among various solvents reported for *T. arjuna* extraction, methanol has demonstrated strong efficacy in recovering phenolics and flavonoids due to its polarity and ability to penetrate plant tissues. Therefore, methanol was selected as the extraction solvent in this study to maximize the recovery of bioactive compounds during ultrasound‐assisted extraction. Polyphenols in *T. arjuna* bark can degrade during the traditional solvent extraction (CSE) technique because of the elevated temperatures, oxidative conditions, and prolonged extraction times. Meanwhile, the ultrasound‐assisted extraction (UAE) demonstrated superior polyphenol extraction from several biomaterials; other variables like solvent type and concentration, temperature, solvent‐to‐solid ratio, and duration of extraction still need to be optimized (Vinatoru et al. 2017). Although the absolute mass yield is a useful practical indicator, the true significance of an optimized extraction process is its ability to enhance the concentration of desired bioactive compounds in the extract, even when the overall yield remains relatively low.

While Ultrasound‐Assisted Extraction (UAE) has been previously implemented to *T. arjuna* (Adeel et al. 2022; Meena et al. 2020), a comprehensive enhancement using Response Surface Methodology (RSM) to model the critical interactive effects of sonication power, temperature, time, and solvent‐to‐solid ratio is lacking. Many existing studies focus primarily on maximizing crude extract yield (Meena et al. 2020; Adeel et al. 2022), without a targeted approach toward obtaining a phytochemically rich profile. Furthermore, a holistic characterization linking the optimized UAE conditions to a detailed phytochemical composition, nutritional value, and resultant antioxidant activity has not been fully explored (Meena et al. 2020). Therefore, the novelty of this study stems from the systematic, RSM‐guided optimization of UAE to produce a qualitatively superior extract, followed by its thorough proximate, mineral, and bioactivity profiling.

This research aimed to (1) Optimize the UAE parameters for T. arjuna bark using a Box–Behnken design to enhance the extraction efficiency of bioactive compounds (2) Characterize the proximate and mineral composition of the bark and (3) Conduct a detailed phytochemical and antioxidant analysis of the extract obtained under the optimized conditions to validate its quality and potential bioactivity.

## 2 Materials and Method

### 2.1 Plant Material

The dried *T. arjuna* bark was sourced from the botanical gardens of Lahore. The plant material was authenticated and a voucher specimen (GCU‐BOT‐23‐3719) was submitted at the Herbarium of the Government College University, Lahore. Collected samples were washed and dried in a Harvest Saver Commercial Dehydrator (R‐5A, Eugene, OR, USA). The plant material was then powdered using an electrical grinder (Silver Crest model no. SC‐350G). The obtained powder was meticulously placed in a refrigerator at 4°C and was used directly for ultrasound‐assisted extraction within 48 h of grinding (Kumar et al. 2018).

### 2.2 Proximate Analysis of *T. Arjuna Bark's Extract*

Using established techniques from the Association of Official Analytical Chemists, the contents of the sample were measured for crude protein, total ash, moisture, crude fiber, crude fat and nitrogen‐free extract (AOAC International 2020).

### 2.3 Mineral Analysis

Elemental analysis of arjuna bark powder was carried out in conformity with established protocol involving the usage of atomic absorption spectrophotometer (AOAC International 2020). Atomic Absorption Spectrophotometer (Model: S2, Make: Thermo, USA) with SOLAAR software was used to determine the minerals cadmium (Cd), chromium (Cr), nickel (Ni), iron (Fe), copper (Cu), calcium (Ca), manganese (Mn), lead (Pb), cobalt (Co), mercury (Hg) and zinc (Zn) contents of the digested solution (Meena et al. 2020).

### 2.4 Ultrasound Assisted‐Methanol Extraction

Ultrasound (model VCX 750, Sonics & Materials Inc., Newtown, CT, USA) technology‐assisted method was utilized for the isolation of *T. Arjuna* bark powder using methanol as the solvent (Adeel et al. 2022). Methanol was selected as the solvent of the extraction for this study due to its well‐established effectiveness in extracting a wide range of medium‐ to high‐polarity bioactive compounds, especially phenolic acids and flavonoids, which are the primary targets of this optimization (Chemat et al. 2017; Tiwari 2015). Its polarity index allows for effective penetration of plant cell walls and solubilization of these target phytochemicals. Furthermore, methanol has been successfully used in previous phytochemical studies on *T. arjuna* bark, demonstrating its ability to yield extracts with significant antioxidant activity, which aligns with the objectives of this research (Daimary 2024; Kumar et al. 2018).

Specimens were measured (100 ± 0.1 g) using a digital balance model Kern 440‐35 N (KERN & Sohn GmbH, Balingen‐Frommern, Germany) during each trial. The impact of temperature (°C), solute to solvent ratio (S:S), sonication power (%), and time (min) was analyzed by implementing the Box–Behnken design for maximum extraction of methanol extraction of arjuna bark samples. Using a digital thermometer, the media's temperature was maintained within ±1.5°C.

### 2.5 Experimental Design

Box–Behnken design for optimal extraction of methanol extraction of arjuna bark was constructed to select the optimized formula for preparation of methanol extract of *T. Arjuna's* bark. Design expert (version 11, stat‐ease, USA) software was utilized in this regard and three levels of each independent factor were used for the optimization. The selected level of different parameters was: Temperature (°C) minimum 40°C and maximum 80°C, Solute to solvent ratio (S:S) minimum 10 and maximum 20, Sonication power (%) minimum 50% and maximum 90% and Time (min) minimum 5 min and maximum 15 min as presented in Tables 1 and 2. The effect of these independent variables was assessed in terms of percentage yield (%).

**TABLE 1:** Independent variables with their coded and actual values for optimizing the yield of *T. arjuna* bark, as determined by the Box–Behnken design.

Factor	Name	Units	−1	0	+1
A	Sonication Power	%	50	70	90
B	Temperature	°C	40	60	80
C	Time	min	5	10	15
D	Solvent/Solute Ratio	S: S	10	15	20

**TABLE 2:** Treatment plan for optimizing the yield of *T. arjuna* bark, as determined by Box–Behnken design.

Run	A: Sonication power	B: Temperature	C: Time	D: Solvent/Solute ratio
	%	°C	Minutes	S: S
1	70	80	10	20
2	70	40	10	20
3	70	60	10	15
4	90	60	10	10
5	50	80	10	15
6	70	40	15	15
7	70	40	10	10
8	70	80	5	15
9	70	60	10	15
10	70	80	10	10
11	90	40	10	15
12	70	60	10	15
13	70	60	15	20
14	70	40	5	15
15	70	60	5	20
16	90	60	5	15
17	50	60	5	15
18	50	60	10	10
19	90	60	10	20
20	70	60	15	10
21	90	80	10	15
22	70	80	15	15
23	90	60	15	15
24	50	60	15	15
25	70	60	10	15
26	70	60	10	15
27	50	60	10	20
28	70	60	5	10
29	50	40	10	15

The four independent variables for UAE: temperature, solvent‐to‐solid ratio, sonication power, and extraction time were determined based on their established significance in the literature concerning plant material extraction (Alam et al. 2020; Habib et al. 2021). The specific levels for each variable were determined based on preliminary experiments and previous studies to identify ranges that would produce a meaningful effect on the extraction yield while operating within practical limits.

- The temperature ranges of 40°C–80°C was chosen to enhance solubility and mass transfer while remaining below the boiling point of methanol (65°C) to minimize solvent loss and potential degradation of thermolabile compounds (Drăghici‐Popa et al. 2025; Khan et al. 2022).
- The solvent‐to‐solid ratio (10:1 to 20:1 mL/g) was selected to ensure sufficient solvent volume for complete immersion and efficient mass transfer without being excessively dilute (Adam‐Dima et al. 2024; Santos et al. 2020).
- Sonication power levels (50%–90%) were set within the effective operational range of the probe to ensure adequate cavitation intensity without causing excessive frothing or temperature spikes.
- The extraction time (5–15 min) was limited to leverage the key advantage of UAE rapid extraction and to prevent potential degradation of compounds over prolonged exposure to ultrasonic energy (Chemat et al. 2017; Tiwari 2015).

The coded and actual values of the independent variables used in the Box–Behnken design are presented in Table 1.

### 2.6 Phytochemical Characterization of the Optimized Methanolic Extract

Analysis for flavonoids, phenols, tannins, and terpenoids was carried out as per standard methods (Qi et al. 2025). The method for phytosteroid quantification was adapted from Kumar et al. (2023) using gas chromatography.

#### 2.6.1 Total Phenolic Content (TPC) Determination

With minor adjustments, the Folin–Ciocalteu colorimetric method (Singleton et al. 1999), as implemented in more recent works (Domínguez‐López et al. 2024; Molole et al. 2022), was used to determine the total phenolic content. 100 μL of Folin–Ciocalteu reagent (diluted 1:10 with distilled water) was combined with 20 μL of the methanolic extract. For 5 min, the solution was kept at room temperature. Thereafter, 80 μL of a 7.5% (w/v) sodium carbonate solution was incorporated. After vortexing, the tubes were kept at room temperature for 2 h in the dark. Following incubation, a microplate reader (or spectrophotometer) was utilized to calculate the absorbance at 765 nm. Gallic acid was used to create a standard curve (0–500 mg/L). The findings were presented as milligrams of gallic acid equivalent (GAE) per gram of dry extract (mg GAE/g extract). Every analysis was carried out three times.

#### 2.6.2 Total Flavonoid Content (TFC) Determination

With a few minor adjustments, the aluminum chloride colorimetric method (Zhishen et al. 1999) as applied in more recent works (Kumar et al. 2018) was followed to measure the total flavonoid content. 150 μL of 95% ethanol was combined with 50 μL of the methanolic extract. Ten microliters of a potassium acetate solution (1 M) and ten microliters of an aluminum chloride (AlCl₃) solution (10%, w/v) were then added. Distilled water was utilized to lower the final volume to 250 μL. After thoroughly mixing the solution in vortex, it was kept at room temperature for half an hour. Following incubation, a microplate reader (or spectrophotometer) was utilized to calculate the absorbance at 415 nm. Quercetin was employed to establish a standard curve (0–500 mg/L). The results were expressed as milligrams of quercetin equivalent (QE) per gram of dry extract (mg QE/g extract). Every analysis was carried out three times.

#### 2.6.3 Quantification of Tannins, Saponins, and Terpenoids

##### 2.6.3.1 Total Tannin Content

The total tannin content was calculated using the Folin‐Denis method. 0.1 mL of extract was combined with 0.5 mL of Folin‐Denis reagent and 1 mL of saturated sodium carbonate solution. Absorbance was recorded at 700 nm after 30 min of incubation. Tannic acid was utilized as a standard, and the results were reported in milligrams of tannic acid equivalents (TAE) per gram of extract (Molnar et al. 2024).

##### 2.6.3.2 Total Saponin Content

The total saponin content was calculated using a gravimetric technique. 5 g of extract was refluxed with 20% aqueous ethanol, and the purified saponins were extracted by successive solvent partitioning with diethyl ether and n‐butanol. The residue was dried to a consistent weight, and the saponin concentration was measured using mg per gram of extract (mg/g extract) (Labu et al. 2025).

##### 2.6.3.3 Total Terpenoid Content

The total terpenoid content was calculated using the Liebermann‐Burchard assay. 0.5 mL of extract was combined with chloroform and then treated with strong sulfuric acid. After 2 h of chromogen development, the absorbance was calculated at 538 nm. Linalool was employed as a standard, and the results are reported as mg linalool equivalents per gram of extract (mg LE/g extract) (Le Bot et al. 2022).

### 2.7 Antioxidant Activity of the Optimized Methanolic Extract

#### 2.7.1 The Ferric Reducing Antioxidant Power (FRAP) Assay

The Benzie and Strain method was utilized to measure the ability to decrease ferric ions. Using the linear calibration plot for FeSO_4_ (2.5–20 μM), the antioxidant capability determined on the extract's capability to eliminate ferric ions was computed and represented as μM FeSO_4_ equivalents per gram of extract (Kumar et al. 2016).

#### 2.7.2 The DPPH (2,2‐Diphenyl‐1‐Picryl‐Hydrazyl) Assay

The extract's capability to scavenge DPPH radicals was estimated using the technique outlined by Barros et al. The formula below was applied to determine the percentage of inhibitory activity:

$$\%\text{Inhibition}=\frac{\mathrm{Ac}-\mathrm{As}}{\mathrm{Ac}}\;\times 100$$

where as is the extract/standard absorbance and Ac is the control absorbance. The requisite quantity of extract or standard needed to stabilize 50% of DPPH radicals was indicated by the IC50 value, which is used to articulate free radical scavenging activities of studied samples (Kumar et al. 2018; Rombaut et al. 2020).

### 2.8 Calculation of Bioactive Compound Yield and Extraction Efficiency

To provide a more comprehensive assessment of the extraction process beyond crude extract mass, the yields of specific bioactive compounds per unit of dry plant material were calculated.

The yield of bioactive components was calculated as follows:

$$\begin{array}{c} \text{Yield}\;\left(\mathrm{mg}\;\text{bioactives}/100\;g\;\text{bark}\right)=\left[\text{Phytochemical Concentration}\right.\\ \;\left.\left(\mathrm{mg}/g\;\text{extract}\right)\right]\times \left[\text{Extract Mass Yield}\right.\\ \;\left.\left(g\;\text{extract}/100\;g\;\text{bark}\right)\right] \end{array}$$

The extraction efficiency rate for phenolics, considering processing time, was determined as:

$$\begin{array}{c} \text{Efficiency}\;\left(\mathrm{mg}/100\;g/h\right)=\left[\text{Phenolic Yield}\;\left(\mathrm{mg}\;\mathrm{GAE}/100\;g\;\text{bark}\right)\right]/\\ \;\left[\text{Extraction Time}\;\left(h\right)\right] \end{array}$$

### 2.9 Statistical Analysis

The Box–Behnken design used for modeling the system's behavior by utilizing a quadratic equation for the specific purpose. Design Expert software package was utilized to evaluate the level of significance of *T. arjuna* bark yield by analysis of data (Suwonsichon 2019). Three sonication trials were conducted for every treatment. Additionally, obtained mean outputs, with the standard deviations, were utilized. A threshold level of 5% was used to assess notable variations between the diverse therapies.

## 3 Results and Discussion

### 3.1 Proximate Analysis of *T. Arjuna* Bark

Proximate analysis of the bark of *T. arjuna* was carried out to investigate its possibilities. Table 3 presents the average compositional values of *T. arjuna*, disclosing its rich nutritional composition, including moisture 11.24% ± 1.30%, ash 14.78% ± 0.08%, fat 1.07% ± 0.15%, crude protein 3.53% ± 0.07%, fiber 14.06% ± 0.23%, and NFE 36.20% ± 1.35%.

**TABLE 3:** Proximate analysis of *T. arjuna's* bark.

Test	Results (%)
Moisture	11.24 ± 1.30
Ash	14.78 ± 0.08
Fat	1.07 ± 0.15
Protein	3.53 ± 0.07
Fiber	14.06 ± 0.23
NFE	36.20 ± 1.35

*Note:* Values are expressed as mean ± SD (*n* = 3).

### 3.2 Mineral Analysis

The mineral analysis of *T. arjuna* bark revealed significant levels of essential minerals, including calcium (86.27 ± 1.43 mg/kg), iron (16.03 ± 1.21 mg/kg), zinc (8.69 ± 1.03 mg/kg), manganese (9.15 ± 1.03 mg/kg), and copper (9.53 ± 1.05 mg/kg). These minerals are vital for various physiological functions, such as bone health, immune response, and antioxidant defense. The high calcium content aligns with results from Meena et al. (2020), who noted that *T. arjuna* bark is a substantial source of calcium, beneficial for managing bone‐related issues like osteoporosis. The iron content further supported its traditional use in treating anemia, as highlighted by Kumar et al. (2023), who emphasized the influence of iron in hemoglobin synthesis and oxygen transport. Additionally, the presence of zinc and copper enhances the bark's therapeutic potential, as these trace elements are crucial for enzymatic activities and immune function. Paul et al. (2022) pointed out that zinc and copper serve as essential cofactors for antioxidant enzymes like superoxide dismutase (SOD), which help reduce oxidative stress, further highlighting the bark's antioxidant properties.

Trace amounts of heavy metals, such as cadmium (0.29 ± 0.12), lead (0.12 ± 0.02), and mercury (0.09 ± 0.06 mg/kg), were also detected but stayed within the established parameters defined by regulatory bodies like the WHO and FAO. Alam et al. (2020) found similar results in their analysis of medicinal plants, confirming that trace heavy metal levels do not undermine the safety or efficacy of plant‐based therapeutics.

### 3.3 Ultrasound‐Assisted Optimal Extraction of *T. Arjuna* Bark

Using a three‐level, five‐factor Box–Behnken design, the influence of multiple parameters such as sonication power (%), solute to solvent ratio (S:S), temperature (°C), and time (min) was investigated. Twenty nine extraction runs in total were performed, as indicated by the Box–Behnken design. This table displays the yields of extract from the various extraction runs. The yield varied from 8.18% ± 0.21% to 12.09% ± 0.28%, as Table 4 clearly shows. The optimization process was crucial for identifying the conditions that maximize the efficiency of phytochemical release from the complex bark matrix. Furthermore, phytochemical analysis confirmed that this optimized extract was abundant in phenolic and flavonoid compounds, demonstrating that the process successfully enhanced the extraction of valuable components. To quantitatively demonstrate this, the yield of key phytochemicals per unit mass of dry bark was calculated. Under the optimized UAE conditions, the process delivered 53.46 mg of gallic acid equivalents (GAE) and 35.62 mg of quercetin equivalents (QE) per 100 g of T. arjuna bark. This represents a highly concentrated extraction of valuable bioactives. When processing time is considered, the UAE process achieved a phenolic extraction rate of 213.84 mg GAE per 100 g bark per hour, an efficiency that far surpasses conventional methods like Soxhlet extraction, which typically operate over much longer durations (6–24 h) for exhaustive mass recovery, often at the cost of higher energy consumption and potential degradation of thermolabile compounds (Tiwari 2015; Vinatoru et al. 2017).

**TABLE 4:** Percentage yield of Arjuna bark extract conducted using the Box–Behnken design.

Run	A: Sonication power	B: Temperature	C: Time	D: Solvent/Solute ratio	Yield
	%	C	Min	S: S	%
1	70	80	10	20	10.24 ± 1.21
2	70	40	10	20	8.91 ± 0.65
3	70	60	10	15	9.74 ± 1.06
4	90	60	10	10	11.41 ± 1.33
5	50	80	10	15	8.98 ± 0.73
6	70	40	15	15	8.74 ± 0.65
7	70	40	10	10	8.51 ± 0.79
8	70	80	5	15	8.69 ± 0.58
9	70	60	10	15	8.55 ± 1.25
10	70	80	10	10	8.47 ± 1.23
11	90	40	10	15	10.91 ± 1.26
12	70	60	10	15	8.85 ± 0.67
13	70	60	15	20	9.21 ± 1.09
14	70	40	5	15	8.42 ± 1.31
15	70	60	5	20	8.52 ± 0.21
16	90	60	5	15	10.37 ± 1.24
17	50	60	5	15	8.27 ± 0.06
18	50	60	10	10	8.18 ± 0.71
19	90	60	10	20	11.19 ± 1.31
20	70	60	15	10	9.11 ± 1.02
21	90	80	10	15	12.09 ± 1.39
22	70	80	15	15	9.78 ± 1.02
23	90	60	15	15	11.81 ± 1.35
24	50	60	15	15	9.39 ± 0.68
25	70	60	10	15	9.41 ± 0.23
26	70	60	10	15	9.12 ± 1.12
27	50	60	10	20	8.82 ± 0.05
28	70	60	5	10	9.01 ± 0.70
29	50	40	10	15	9.02 ± 1.03

*Note:* Values are expressed as mean ± SD (*n* = 3).

While the absolute extraction yields were modest (ranging from 8.18% to 12.09%), the optimization process was crucial for identifying the conditions that maximize the efficiency of phytochemical release from the complex bark matrix. Furthermore, phytochemical analysis confirmed that this optimized extract was abundant in phenolic and flavonoid compounds, demonstrating that the process successfully enhanced the extraction of valuable components.

The present study achieved an optimized extraction yield of 12.09% for *T. arjuna* bark using UAE. While this yield may appear modest when considered in isolation, its significance becomes clear when evaluating the process's efficiency and quality of the extract gained. Ultrasound‐assisted extraction is renowned for its capability to destroy cell barriers more effectively than traditional methods, not necessarily to extract a larger total mass of material, but to do so more rapidly and with higher selectivity for intracellular compounds (Labu et al. 2025).

When compared to conventional methods like Soxhlet extraction, which often requires high temperatures (≥ 60°C) and extended extraction times (6–24 h), the optimized UAE process in this study was completed in a maximum of 15 min (Chemat et al. 2017). Although Soxhlet might produce a higher absolute yield due to exhaustive extraction, it often results in the degradation of thermolabile bioactive compounds and requires significantly more energy and solvent (Domínguez‐López et al. 2024). The value of the UAE process lies in its ability to achieve a high yield of target phytochemicals per gram of extract in a fraction of the time, making it a greener and more efficient alternative. This study focused on optimizing for this efficient release of valuable components, and subsequent phytochemical analysis confirms that the extract is indeed rich in potent compounds, justifying the approach.

### 3.4 Bioactive‐Based Yield and Process Efficiency

While the absolute mass yield of the crude extract provides one measure of process performance, a more relevant metric for evaluating an optimized extraction is the effective yield of the target bioactive compounds (Chemat et al. 2017; Zhang et al. 2018). To quantitatively demonstrate this, the yield of key phytochemicals per unit mass of dry starting material was determined as outlined in the Materials and Methods section. Under the optimized UAE conditions (Run 21: 90% power, 80°C, 10 min, 15:1 ratio), which yielded 12.09% crude extract, the process delivered 53.46 mg of gallic acid equivalents (GAE) and 35.62 mg of quercetin equivalents (QE) per 100 g of *T. arjuna* bark. This represents a highly concentrated extraction of valuable bioactives. Furthermore, when processing time is considered, the UAE process achieved a phenolic extraction rate of 213.84 mg GAE per 100 g bark per hour an efficiency that far surpasses conventional methods like Soxhlet extraction, which typically operate over much longer durations (6–24 h) for exhaustive mass recovery, often at the cost of higher energy consumption and potential degradation of thermolabile compounds (Tiwari 2015; Vinatoru et al. 2017). This demonstrates that the optimized UAE process successfully prioritizes the rapid and selective release of target phytochemicals over the exhaustive extraction of all soluble solids, resulting in a potent, high‐quality extract.

### 3.5 Fitting the Experimental Model

The analysis of the Lack of Fit *F*‐value offers insights into how well the model captures the underlying link between the factors and the response variable. In this instance, the Lack of Fit *F*‐value is 0.73, accompanied by a *p*‐value of 0.6896, indicating that the lack of fit is not statistically significant at conventional levels (e.g., *α* = 0.05). This explains that the model positions with the observed data sufficiently, with any deviations likely attributed to random variation rather than a systematic flaw in the model's structure (Table 5). According to (Montgomery 2017), a nonsignificant Lack of Fit F‐value is favorable, as it depicts that the predictions of the model are consistent with the experimental data and that there is no strong indication of missing important terms or relationships. However, it is crucial to recognize that the reliability of the Lack of Fit test depends on having replicated observations in the experimental design to ensure an accurate estimate of pure error (Myers et al. 2016). The high probability (68.96%) that the observed Lack of Fit F‐value results from random variation further reinforces the conclusion that the model is suitable for the data as shown in Equation (1). Nevertheless, it is advisable to validate the model using additional diagnostic tools, such as residual analysis and cross‐validation, to ensure its robustness and generalizability (Myers et al. 2016).

**TABLE 5:** Interpretation of variance (ANOVA) for *T. arjuna* extract.

Source	Sum of squares	df	Mean square	*F*	*p*	
Model	31.36	14	2.24	12.85	0.0123	Significant
A‐Sonication Power	19.05	1	19.05	109.32	< 0.0001	Significant
B‐Temperature	1.17	1	1.17	6.69	0.0215	Significant
C‐Time	1.89	1	1.89	10.83	0.0054	Significant
D‐Solvent/Solute Ratio	0.4033	1	0.4033	2.31	0.1504	Nonsignificant
AB	0.3721	1	0.3721	2.14	0.1660	Nonsignificant
AC	0.0256	1	0.0256	0.1469	0.7073	Nonsignificant
AD	0.1849	1	0.1849	1.06	0.3205	Nonsignificant
BC	0.1482	1	0.1482	0.8505	0.3720	Nonsignificant
BD	0.4692	1	0.4692	2.69	0.1231	Nonsignificant
CD	0.0870	1	0.0870	0.4994	0.4914	Nonsignificant
A^2^	6.31	1	6.31	36.18	< 0.0001	Significant
B^2^	0.0044	1	0.0044	0.0250	0.8766	Nonsignificant
C^2^	0.1540	1	0.1540	0.8837	0.3631	Nonsignificant
D^2^	0.0959	1	0.0959	0.5502	0.4705	Nonsignificant
Residual	2.44	14	0.1743			
Lack of Fit	1.57	10	0.1574	0.7278	0.6896	Nonsignificant
Pure Error	0.8653	4	0.2163			
Cor Total	33.80	28				

#### 3.5.1 Final Equation With Respect to Coded Factors

(1) $$\begin{array}{c} Y=+9.13+1.26A+0.3117B+0.3967C+0.1833D0.3050\mathrm{AB}\\ \;+0.0800\mathrm{AC}-0.2150\mathrm{AD}+0.1925\mathrm{BC}+0.3425\mathrm{BD}\\ \;+0.1475\mathrm{CD}+0.9859A^{2}+0.0259B^{2}-0.1541C^{2}-0.1216D^{2} \end{array}$$

where A = Sonication Power (W), B = Temperature (°C), C = Time (min), D = Solvent/Solute Ratio (S:S).

Likewise, the second‐order equations derived for *T. Arjuna bark*'s yield in terms of actual variables are as follows.

#### 3.5.2 Conclusive Equation Expressed in Terms of Actual Factors

Y = 19.67331 −0.303571 Sonication Power −0.116192 Temperature −0.057400 Time +0.068567 Solvent/Solute Ratio +0.000763 Sonication Power × Temperature +0.000800 Sonication Power × Time −0.002150 Sonication Power × Solvent/Solute Ratio +0.001925 Temperature × Time +0.003425 Temperature × Solvent/Solute Ratio +0.005900Time × Solvent/Solute Ratio +0.002465 SonicationPower^2^ +0.000065 Temperature^2^−0.006163 Time^2^−0.004863 Solvent/Solute Ratio^2^.

### 3.6 Single Factor Analysis

Effects of temperature (°C), solute to solvent ratio (S:S), time (min), and sonication power (%) on the yield were calculated by controlling each extraction variable's level between −1 and +1 in this study. The sonication power (%) demonstrated some positive effects on the methanolic extract of *T. arjuna* bark. As the sonication power increased from 50% to 90%, the yield consistently rose. The peak yield was recorded at 90% sonication power, suggesting that greater energy input enhances the extraction of bioactive compounds by improving cavitation effects. A previous study reported that ultrasonic treatment served as an environmentally friendly tool for the isolation of arjuna bark from the fabrics of cotton. The same study also demonstrated the method to be effective for the extraction of natural colorants with enhanced strength (Habib et al. 2021). Another study reported that high sonification power increased the extraction of nanoparticles containing a high amount of methanolic extract from the samples of *Terminalia arjuna* (Adeel et al. 2019).

Findings of this study indicated that with a rise in sonication power and temperature, a rise in the trend of yield was also observed. The temperature (°C) exhibits promising authority on the extraction during sonication. Keeping all the other parameters of extraction constant at the average values, an upward trend was observed with increasing temperature (°C) and sonication power (%). Temperature also significantly influenced the extraction process. As the temperature raised from 40°C to 80°C, the yield gradually increased, reaching its peak at 80°C. This indicates that increasing the temperature enhances the solubility of phytochemicals and improves mass transfer rates. No elevated vapor pressure was observed by elevating the temperature (°C) during assisted extraction with ultrasound; subsequently, a reduction in cavitation force would ultimately result in a reduced yield. Those findings indicated distinctively the promising influence of temperature (°C) during sonication on the process of extraction. Furthermore, another study reported a similar sort of findings where high temperature during the process of extraction improved the yield of arjuna bark (Desai et al. 2021). The duration of extraction had a moderate effect on the yield. As the extraction time was extended from 5 min to 15 min, the yield increased, peaking at 15 min. This explains that longer extraction times facilitate better solvent diffusion into the plant matrix and enhance the release of bioactive compounds. The solute‐to‐solvent ratio had a less considerable effect compared to the other factors. However, a slight increase in yield was noted as the ratio rose from 10 to 20, with the highest yield occurring at a ratio of 20. This indicates that a higher solvent volume relative to the solute enhances extraction efficiency by offering a greater driving force for mass transfer. A study reported the extraction of a high yield of argunolic acid from the arjuna bark at high temperature processing (Bharti 2021). However, another study on solute‐solvent extraction regarding arjuna bark revealed that solvent extraction in combination with orbital shaking and refluxing led to better yield production (Adeel et al. 2022). The same research also recommended the utilization of the bark powder for the formulation of medicated supplements, feed for fish and livestock.

### 3.7 Analysis of Mutual Interaction Effect

**FIGURE 1:**
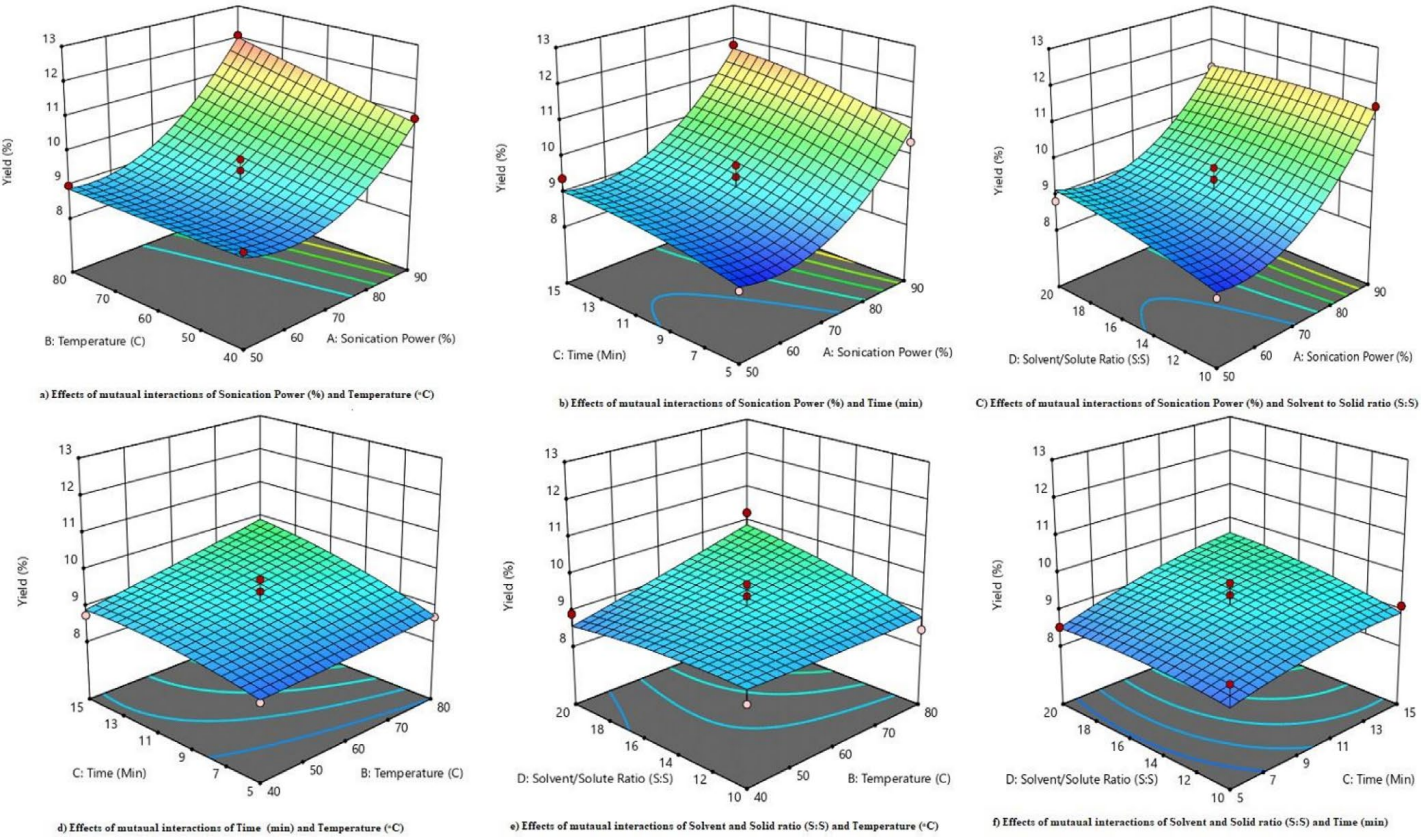
Effects of mutual interactions on yield (%) of *Terminalia arjuna* extract.

The Box–Behnken design was utilized to find the percentage yield on four different factors such as sonication power (%), temperature (°C), time (min) and solute‐solvent ratio (S:S). Analysis of the yield proved to be challenging with mutual factors affecting the yield. The mutual factor analysis illustrated the minimum yield (8.18%) at a temperature (60°C), time (10 min), sonication power (50%) and solute‐solvent ratio. The yield percentage of the *T. arjuna's* bark demonstrated its maximum value at high sonication power with high temperature. Increasing the values of the independent factors showed an increasing trend of the percentage yield. However, decreasing the values of mutual factors had an impact on overall yield, ultimately decreasing its efficiency in the end as shown in Figure 1.

Combining higher sonication power with elevated temperature (Sonication Power × Temperature) significantly increased the yield. The peak extraction efficiency was achieved at 90% sonication power and 80°C, driven by enhanced cavitation and improved solubility of phytochemicals as shown in Figure 1a. Adeel et al. (2022) found that ultrasound‐assisted extraction at high power and temperature notably boosted the extraction yield of *Terminalia arjuna* bark by facilitating cell wall rupture and mass transfer.

Increasing sonication power while extending the extraction time to 15 min (Sonication Power × Time) further enhanced the yield. However, excessive extraction times beyond the optimal duration could reduce cavitation efficiency due to bubble coalescence as shown in Figure 1b. Kumar et al. (2018) highlighted that prolonged sonication times might result in the deterioration of bioactive compounds, ultimately decreasing overall efficiency.

A higher solvent‐to‐solvent ratio of 20:1, in conjunction with high sonication power (Sonication Power × Solvent‐Solute Ratio) at 90%, improved mass transfer as shown in Figure 1c. Alam et al. (2020) that increasing the solvent ratio aids in dissolving bioactive compounds, thus enhancing yield. However, excessively high ratios can dilute the extract, slightly reducing efficiency. Meena et al. (2020) also noted that an optimal solvent‐to‐solute ratio is essential to avoid dilution and ensure effective extraction. These findings are mentioned in Table 6.

**TABLE 6:** Mineral analysis of *T. arjuna* bark.

Minerals	Quantity (mg/Kg)
Calcium (Ca)	86.27 ± 1.43
Cadmium (Cd)	0.29 ± 0.12
Chromium (Cr)	0.82 ± 0.08
Copper (Cu)	9.53 ± 1.05
Iron (Fe)	16.03 ± 1.21
Lead (Pb)	0.12 ± 0.02
Nickel (Ni)	0.59 ± 0.04
Manganese (Mn)	9.15 ± 1.03
Mercury (Hg)	0.09 ± 0.06
Cobalt (Co)	2.07 ± 0.24
Zinc (Zn)	8.69 ± 1.03

*Note:* Values are expressed as mean ± SD (*n* = 3).

Increased temperature (80°C) and extended extraction time (15 min) (Temperature × Time) worked synergistically to boost yield by accelerating solvent diffusion and compound release as shown in Figure 1d. However, Santos et al. (2020) noted that prolonged heating at high temperatures could degrade heat‐sensitive phytochemicals, compromising overall yield quality. A higher solvent volume with a 20:1 ratio at 80°C maximized yield (Temperature × Solvent‐Solute Ratio) by enhancing solute solubility as shown in Figure 1e. Das et al. (2023) indicated that elevated temperatures combined with an optimal solvent ratio significantly improved extraction efficiency, while lower ratios (10:1) led to incomplete extraction due to inadequate solvent availability.

A longer extraction time of 15 min with a high solvent ratio of 20:1 (Time × Solvent‐Solute Ratio) allowed for more effective solvent penetration into the plant matrix, releasing a greater quantity of bioactive compounds as shown in Figure 1f. Sonu Bharti (2021) demonstrated that extended extraction time enhances phytochemical release, although excessive durations can lead to compound degradation. Conversely, shorter extraction times of 5 min with lower ratios resulted in suboptimal yields. Desai et al. (2021) confirmed that insufficient extraction time and low solvent ratios could lead to poor extraction efficiency and reduced bioactive content. These findings underscore the substantial impact of the interactions between variables on extraction efficiency.

### 3.8 Phytochemical Characterization of the Optimized Methanol Extract

Phytochemical studies revealed that the methanol extract of *T. arjuna* bark comprises a diverse span of bioactive compounds, for example phenolics, flavonoids, tannins, terpenoids, saponins, and phyto‐steroids, which have been reported to contribute to its medicinal qualities. To optimize the extraction process, a Box–Behnken design was utilized, resulting in a total of 29 sonication extraction experiments aimed at identifying the ideal conditions for maximizing the yield of these bioactive components (Table 7).

**TABLE 7:** Phytoconstituents present in methanolic extract of arjuna bark.

S. No	Phytoconstituents	Methanol extract (Mean ± SD)
1	Tannins	127.6 ± 23.1 mg TAE/g extract
2	Phenolics	442.2 ± 16.2 mg GAE/g extract
3	Flavonoids	294.6 ± 8.1 mg QE/g extract
4	Terpenoids	3.1 ± 6.8 mg LE/g extract
5	Saponin	0.19 ± 0.02 mg/g extract
6	Phytosteroids	5.7 ± 3.4 mg/g extract

*Note:* Values are provided as mean ± SD (*n* = 3).

Abbreviations: GAE, gallic acid equivalent; LE, linalool equivalent; QE, quercetin equivalent; TAE, tannic acid equivalent.

The phytochemical investigation of the methanol extract showed a notable amount of phenolic and flavonoids, with phenolics quantified at 442.2 ± 16.2 mg GAE/g extract and flavonoids at 294.6 ± 8.1 mg QE/g extract. These bioactive constituents are well known for their potent antioxidant properties, suggesting that the extract may effectively eliminate free radicals and mitigate oxidative stress. A study conducted by Daimary (2024) also highlighted the abundance of phenols and flavonoids that are included in the methanol extract of Arjuna bark. The high concentration of these constituents highlights the extract's potential therapeutic applications, including anti‐inflammatory and cardio protective effects.

Additionally, tannins are present at 127.6 ± 23.1 mg TAE/g extract, contributing to the extract's antioxidant and antimicrobial properties. In contrast, terpenoids (3.1 ± 6.8 mg LE/g extract), saponins (0.19 ± 0.02 mg/g), and phytosteroids (5.7 ± 3.4 mg/g extract) are found in lower concentrations. Although these compounds are secondary constituents, they are still significant due to their bioactivities, such as anti‐inflammatory and cholesterol‐lowering effects and immunomodulator. The presence of these constituents is also confirmed by Kumar et al. (2023). Overall, this phytochemical profile positions the methanol extract as an abundant source of natural antioxidants and a bioactive rich source of natural antioxidants and bioactive substances, with promising applications in healthcare and illness management.

The high concentration of the specific phytochemicals quantified in this extract particularly flavonoids (294.6 mg QE/g) and phenolics (442.2 mg GAE/g) provides a clear chemical rationale for the potent in vitro antioxidant activity observed in the DPPH and FRAP assays. These compound classes are well‐established in the literature as primary contributors to free radical scavenging and metal‐reducing capabilities (Kumar et al. 2023, 2018). Specifically, the high flavonoid content is of significance, as these compounds are known to donate hydrogen atoms, thereby scavenging free radicals. Like DPPH•, which aligns perfectly with our low IC_50_ value of 186.4 μg/mL in the DPPH assay (Kumar et al. 2016).

Furthermore, the considerable tannin content (127.6 mg TAE/g) likely synergizes with the phenolics and flavonoids, enhancing the overall antioxidant capacity of the extract and potentially contributing to antimicrobial properties (Awad et al. 2022). While the concentrations of terpenoids and saponins were lower, their presence is notable. Triterpenoids, such as those found in *T. arjuna*, have been directly linked to the plant's renowned cardioprotective and antidyslipidemic effects, while saponins are known for their hemolytic and immunomodulatory activities (Kumar et al. 2023; Ramesh and Palaniappan 2023).

Therefore, the antioxidant activity demonstrated in this study is not an isolated finding but a direct consequence of the high concentration of these specific bioactive compounds. This in vitro antioxidant potential is a fundamental mechanism underpinning many of the in vivo therapeutic effects attributed to *T. arjuna*, such as cardioprotection (combating oxidative stress in cardiovascular tissues) and anti‐inflammatory activity (scavenging ROS involved in inflammation pathways) (Santos et al. 2020; Paul et al. 2022). While this study confirms the extract's antioxidant foundation, further cell‐based and animal studies are recommended to directly validate these specific bioactivities.

### 3.9 Antioxidant Activity of the Optimized Methanolic Extract

#### 3.9.1 DPPH Free Radical Scavenging Assay

**FIGURE 2:**
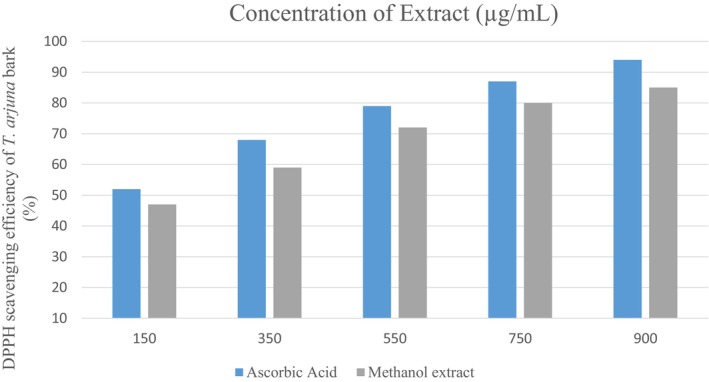
DPPH scavenging efficiency of *Terminalia arjuna* bark methanolic extract.

Figure 2 depicts the dose‐dependent DPPH radical scavenging efficacy of the comparison of the *T. arjuna* bark's methanolic extract with ascorbic acid. The scavenging activity escalated significantly with increasing concentrations for both samples, with ascorbic acid exhibiting marginally greater activity across all evaluated values. At a concentration of 900 μg/mL, the methanolic extract demonstrated roughly 85% scavenging efficacy, whereas ascorbic acid achieved around 95%. The findings indicate a significant antioxidant capacity of the *T. arjuna* bark's methanolic extract, but marginally inferior to that of the standard.

Prior research corroborates these findings. Kumar et al. (2016) documented significant DPPH activity in T. arjuna bark, with the ethanolic extract of the bark exhibiting superior activity (IC50 = 17.41 μg/mL) compared to the leaf extract (IC50 = 20.22 μg/mL). The FRAP experiment also demonstrated more antioxidant activity in the bark (IC50 = 4.781 μM Fe (II) equivalents) than in the leaves (IC50 = 7.572 μM Fe (II) equivalents). Chauhan et al. (2019) noted substantial DPPH radical scavenging capability in T. arjuna fruit extract, emphasizing the plant's antioxidant abundance.

#### 3.9.2 FRAP Assay

The FRAP activity in the T. arjuna bark's methanolic extract was more than that of standard ascorbic acid (Table 8). The bark extract demonstrated an IC₅₀ value of 24.4 μM Fe (II) equivalents, signifying a superior ferric reduction antioxidant capacity compared to ascorbic acid, which had an IC₅₀ of 33.3 μM Fe (II) equivalents. These results correspond with those of Kumar et al. (2018), who examined the antioxidant potential of T. arjuna bark by DPPH and FRAP assays, revealing significant activity in both tests. Khatri et al. (2017) revealed in a related investigation that the *T. arjuna* leaves' methanolic extract had a notable ferric reducing ability, with an EC_1_ value of 232 μg/mL, thus reinforcing the antioxidant potential of different plant components.

**TABLE 8:** Half‐maximal inhibitory concentration (IC_50_) (μg/mL).

Antioxidant assay	Half‐maximal inhibitory concentration (IC_50_) (μg/mL)	
	Ascorbic acid	Methanol extract
DPPH	126.5	186.4
FRAP	33.3 μM Fe (II) equivalents	24.4 μM Fe (II) equivalents

## 4 Conclusion

The present study achieved successful optimization of the ultrasound‐assisted extraction (UAE) of *Terminalia arjuna* bark, applying a Box–Behnken design to systematically analyze critical parameters such as temperature, solvent ratio, sonication power, and extraction duration. The novel focus was not merely on maximizing crude yield, but on efficiently producing a phytochemically rich extract. The application of Response Surface Methodology (RSM) provided unique insights into the interaction of these process variables. Even though the total mass yield of the crude extract was not exhaustive, the optimized extract demonstrated a substantial concentration of phenolic and flavonoid compounds. This phytochemical profile directly correlated with significant in vitro antioxidant potential, as evidenced by strong activity in both DPPH and FRAP assays. The comprehensive analysis confirms that these beneficial polyphenols (including flavonoids and tannins) are linked to a range of therapeutic properties, such as antidyslipidemic, hypocholesterolemic, antioxidant, anticancer, and antibacterial activities. The research underscores that UAE is a rapid, efficient, and green alternative for producing a potent extract. More importantly, it highlights that the quality and bioactivity of an extract are more significant indicators of extraction success than the total mass of crude material obtained. This represents a significant addition to the literature on *T. arjuna* and advances its potential as a source for natural plant‐based therapeutics. Finally, it is essential to note that the promising antioxidant activity was determined through in vitro chemical assays. Further research using cell‐based or animal models is necessary to confirm these bioactive effects within a biological system.

## Author Contributions

Hafsa Tahir contributed to the conceptualization and methodology of the paper. Muhammad Nadeem Akhtar did the data curation and formal analysis. Hamid Saeed has done validation of the data. Muhammad Jawad did the visualization.

## Funding

The authors have nothing to report.

## Disclosure

The authors have nothing to report.

## Consent

All authors agree to publish.

## Conflicts of Interest

The authors declare no conflicts of interest.

## Data Availability

The authors assert that the data supporting the findings of this study is available inside the report.
